# Erratum: Virome composition in marine fish revealed by meta-transcriptomics

**DOI:** 10.1093/ve/veab035

**Published:** 2021-06-17

**Authors:** Jemma L Geoghegan, Francesca Di Giallonardo, Michelle Wille, Ayda Susana Ortiz-Baez, Vincenzo A Costa, Timothy Ghaly, Jonathon C O Mifsud, Olivia M H Turnbull, David R Bellwood, Jane E Williamson, Edward C Holmes

*Virus Evolution*, Volume 7, Issue 1, January 2021, veab005 https://doi.org/10.1093/ve/veab005

Published: 4 February 2021

In the Abstract of this paper a member of the *Matonaviridae* is incorrectly referred to as a “single-strand, negative-sense RNA virus” when the text should read “single-strand, positive-sense RNA virus”.

