# Supplementary material for: Virome composition in marine fish revealed by meta-transcriptomics
Source: Virus Evol. 2021 Feb 4;7(1):veab005. doi: 10.1093/ve/veab005 (PMC7887440; doi:10.1093/ve/veab005)
Supplement: veab005_Supplementary_Data [file veab005_supplementary_data.zip › Geoghegan.SupplementaryFigure1.pdf]

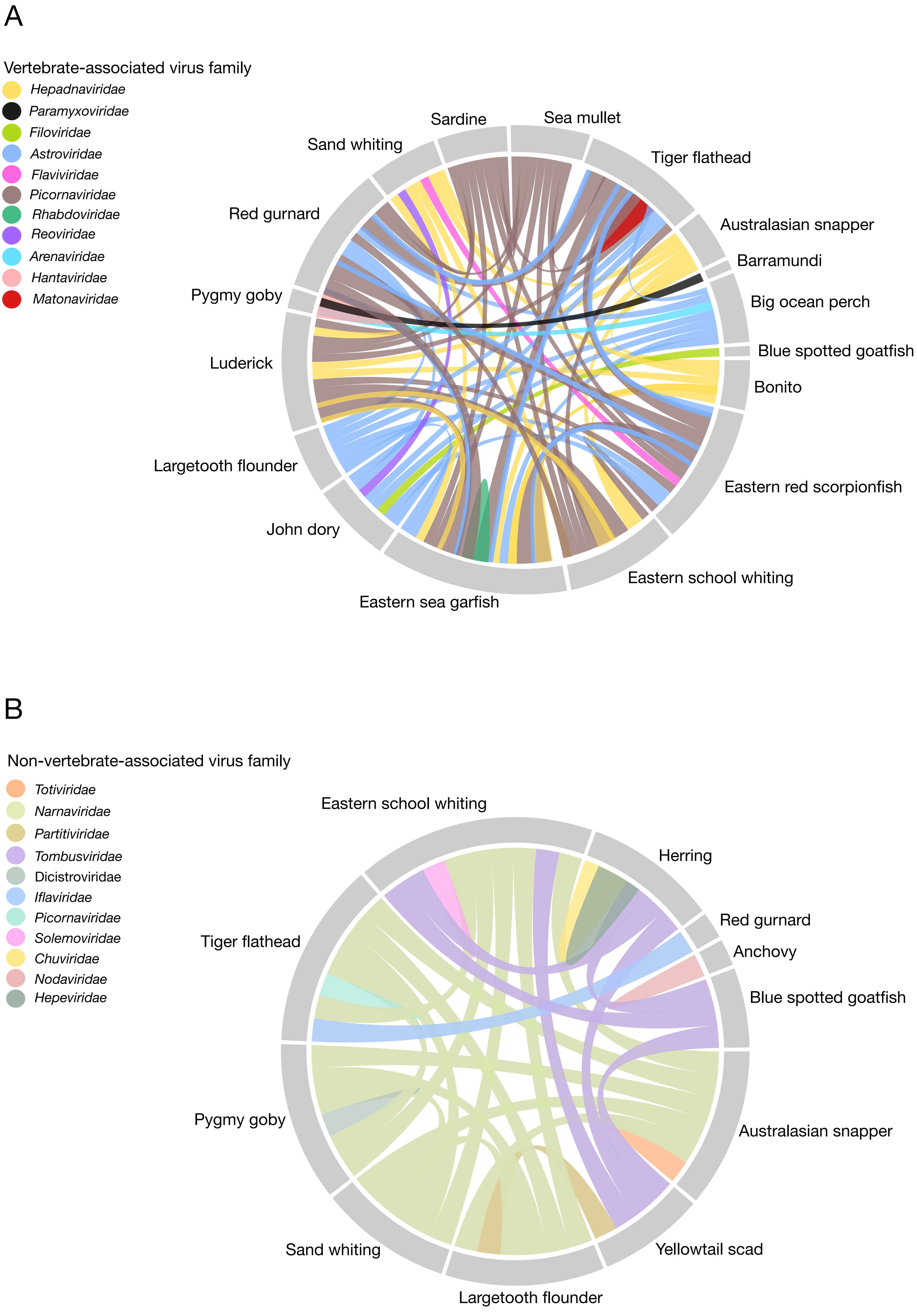

A
Vertebrate-associated virus family
Hepadnaviridae
Paramyxoviridae
Filoviridae
Astroviridae
Flaviviridae
Picornaviridae
Rhabdoviridae
Reoviridae
Arenaviridae
Hantaviridae
Matonaviridae
Sardine
Sea mullet
Sand whiting
Tiger flathead
Red gurnard
Australasian snapper
Barramundi
Big ocean perch
Pygmy goby
Blue spotted goatfish
Luderick
Bonito
Largetooth flounder
Eastern red scorpionfish
John dory
Eastern school whiting
Eastern sea garfish
B
Non-vertebrate-associated virus family
Totiviridae
Narnaviridae
Partitiviridae
Tombusviridae
Dicistroviridae
Iflaviridae
Picornaviridae
Solemoviridae
Chuviridae
Nodaviridae
Hepeviridae
Eastern school whiting
Herring
Red gurnard
Anchovy
Tiger flathead
Blue spotted goatfish
Pygmy goby
Australasian snapper
Sand whiting
Yellowtail scad
Largetooth flounder
