# Supplementary figures and images for: Virome composition in marine fish revealed by meta-transcriptomics
Source: Virus Evol. 2021 Feb 4;7(1):veab005. doi: 10.1093/ve/veab005 (PMC7887440; doi:10.1093/ve/veab005)

# Totiviridae

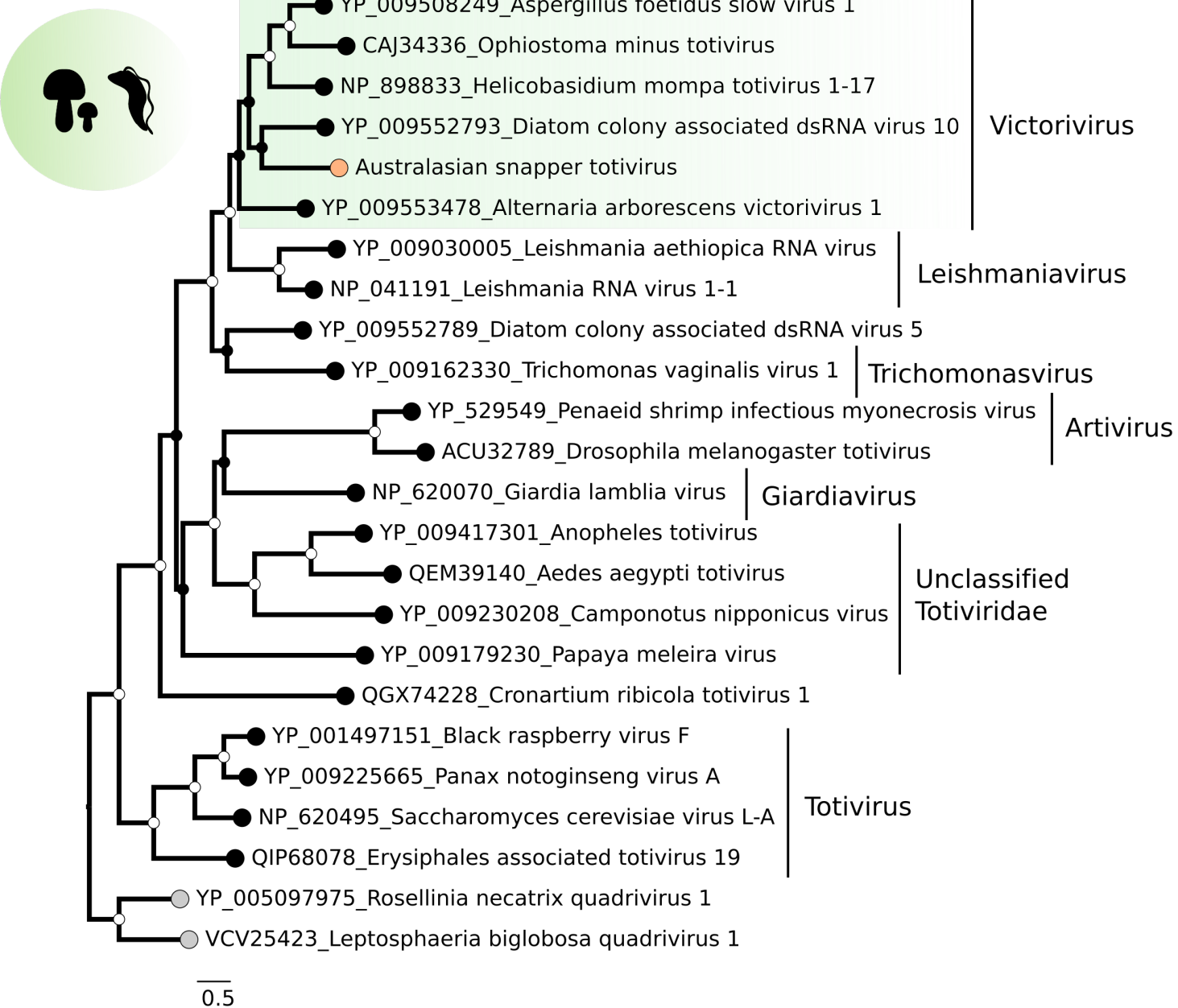

# Narnaviridae

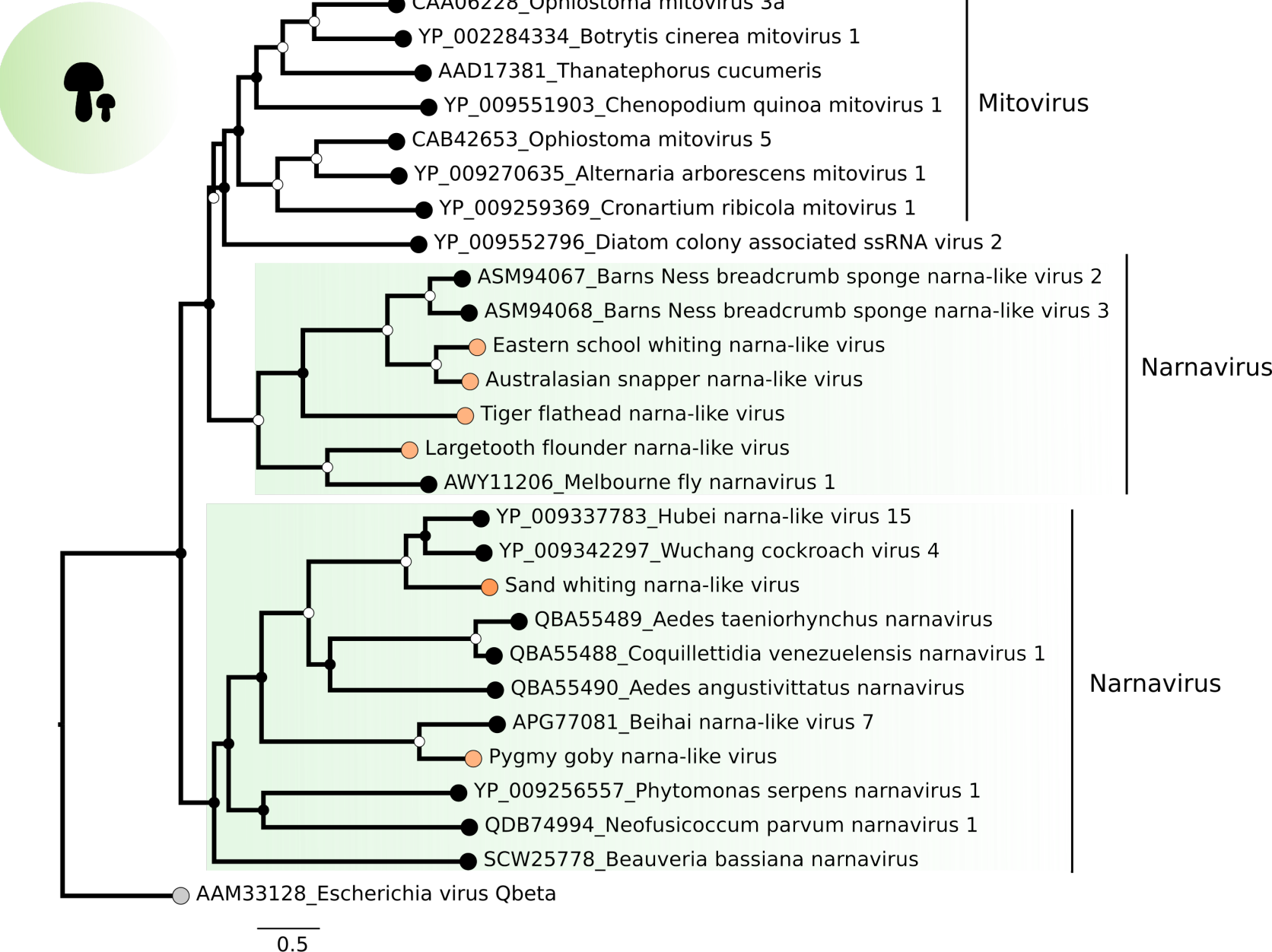

# Partitiviridae

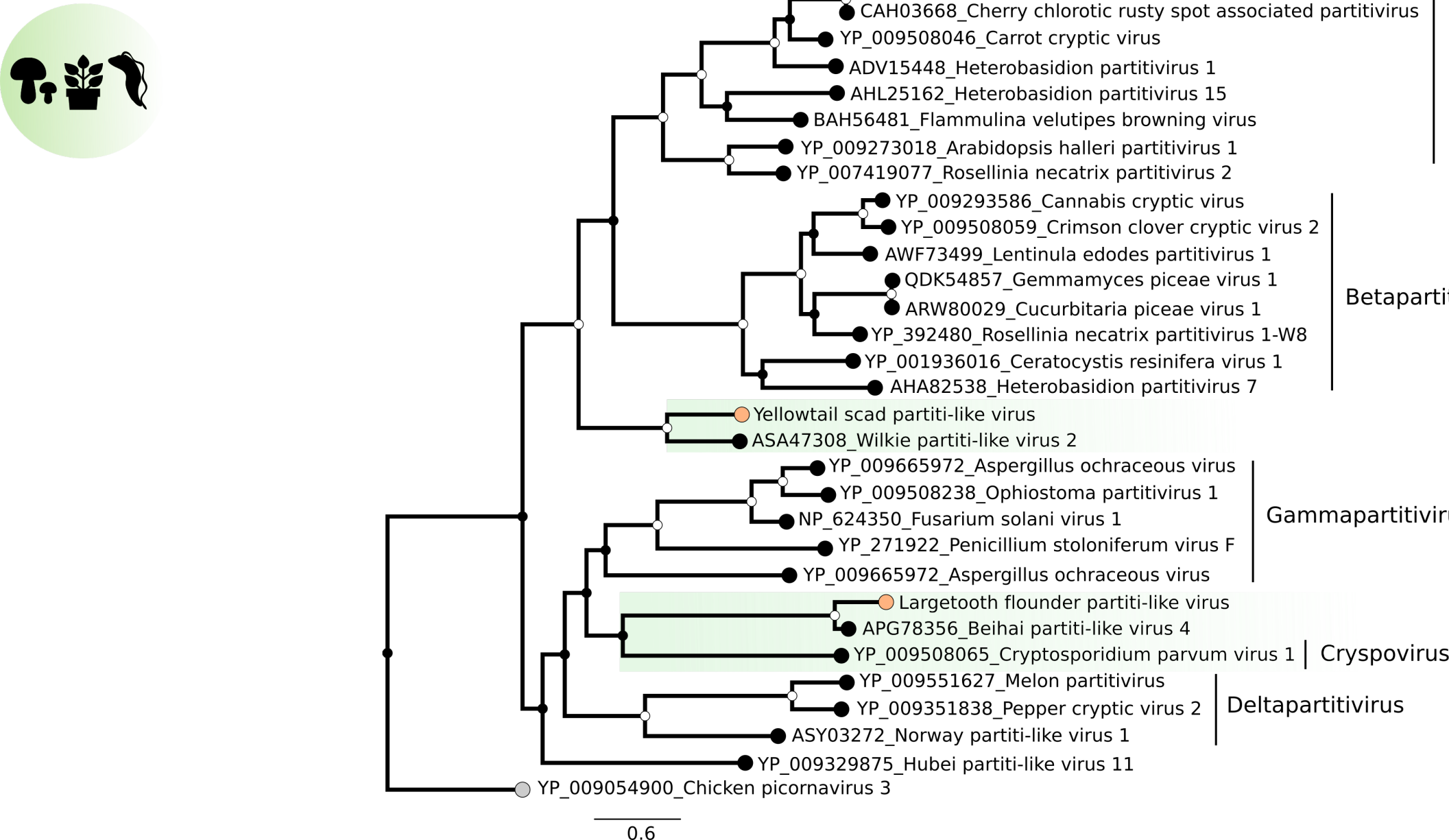

Supplement: veab005_Supplementary_Data [file veab005_supplementary_data.zip › Geoghegan.SupplementaryFigure2.pdf]

# Solemoviridae

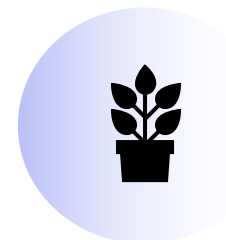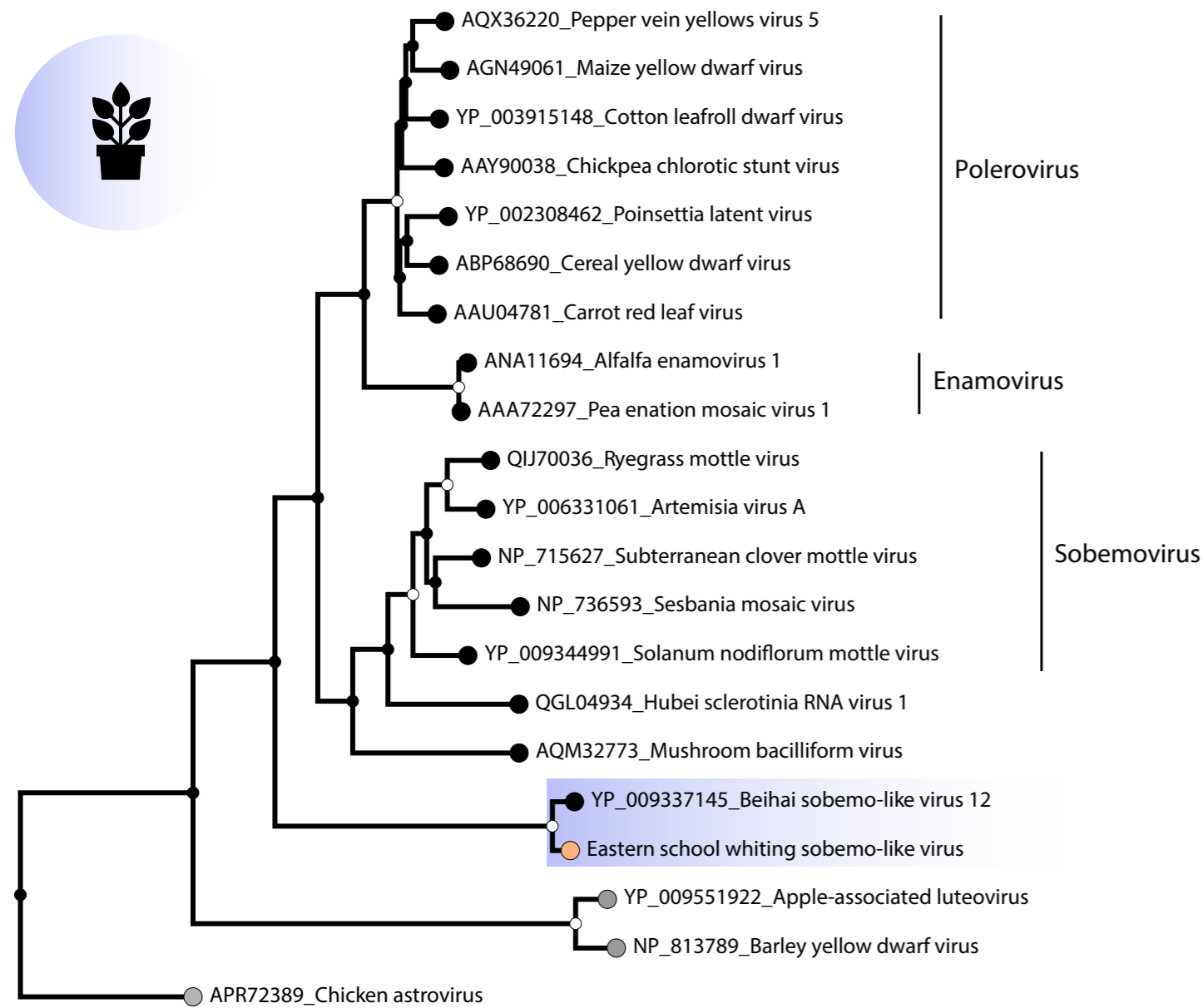

# Tombusviridae

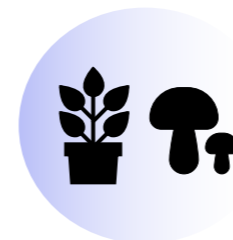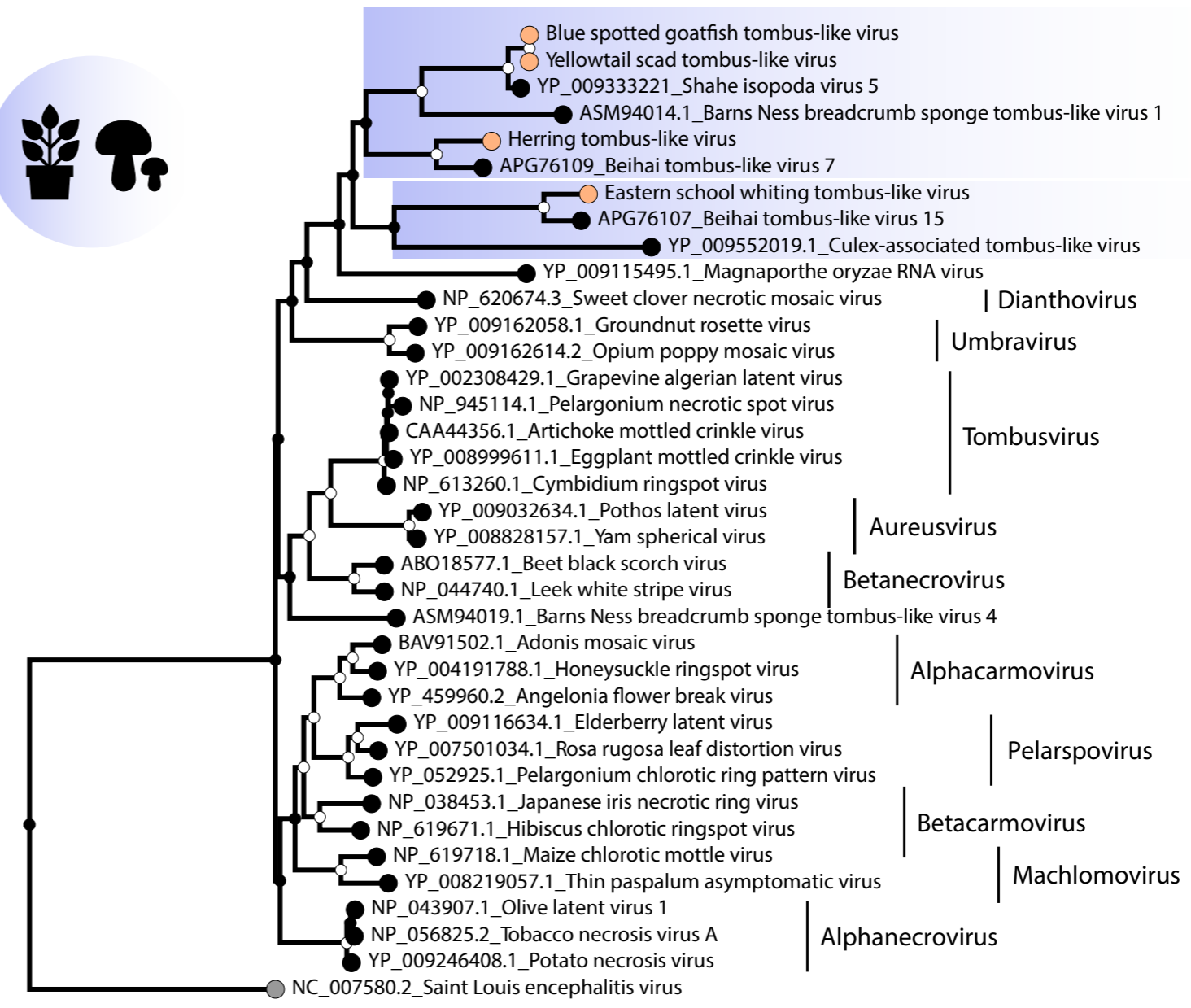

Supplement: veab005_Supplementary_Data [file veab005_supplementary_data.zip › Geoghegan.SupplementaryFigure3.pdf]

## Hepeviridae

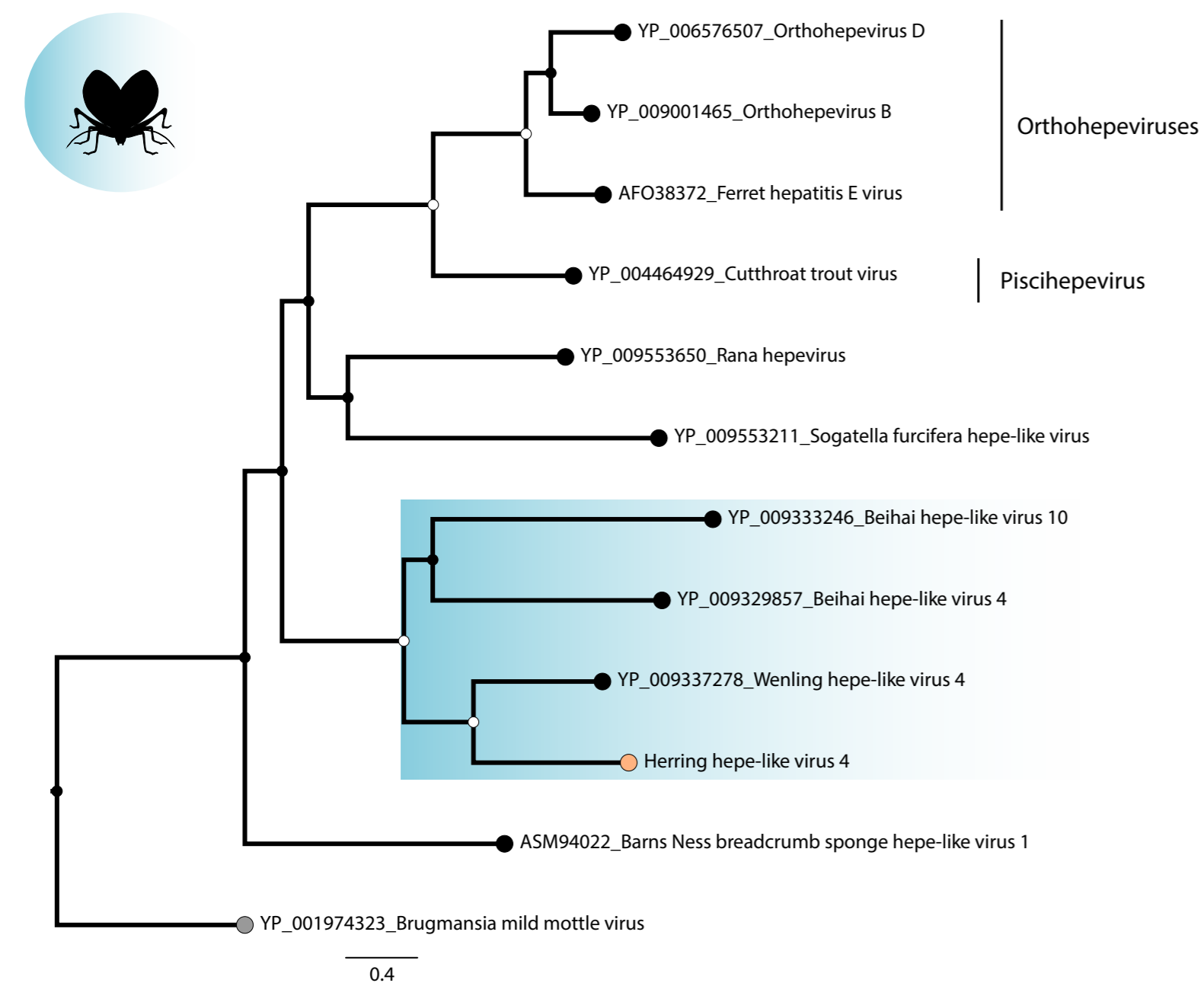

## Chuviridae

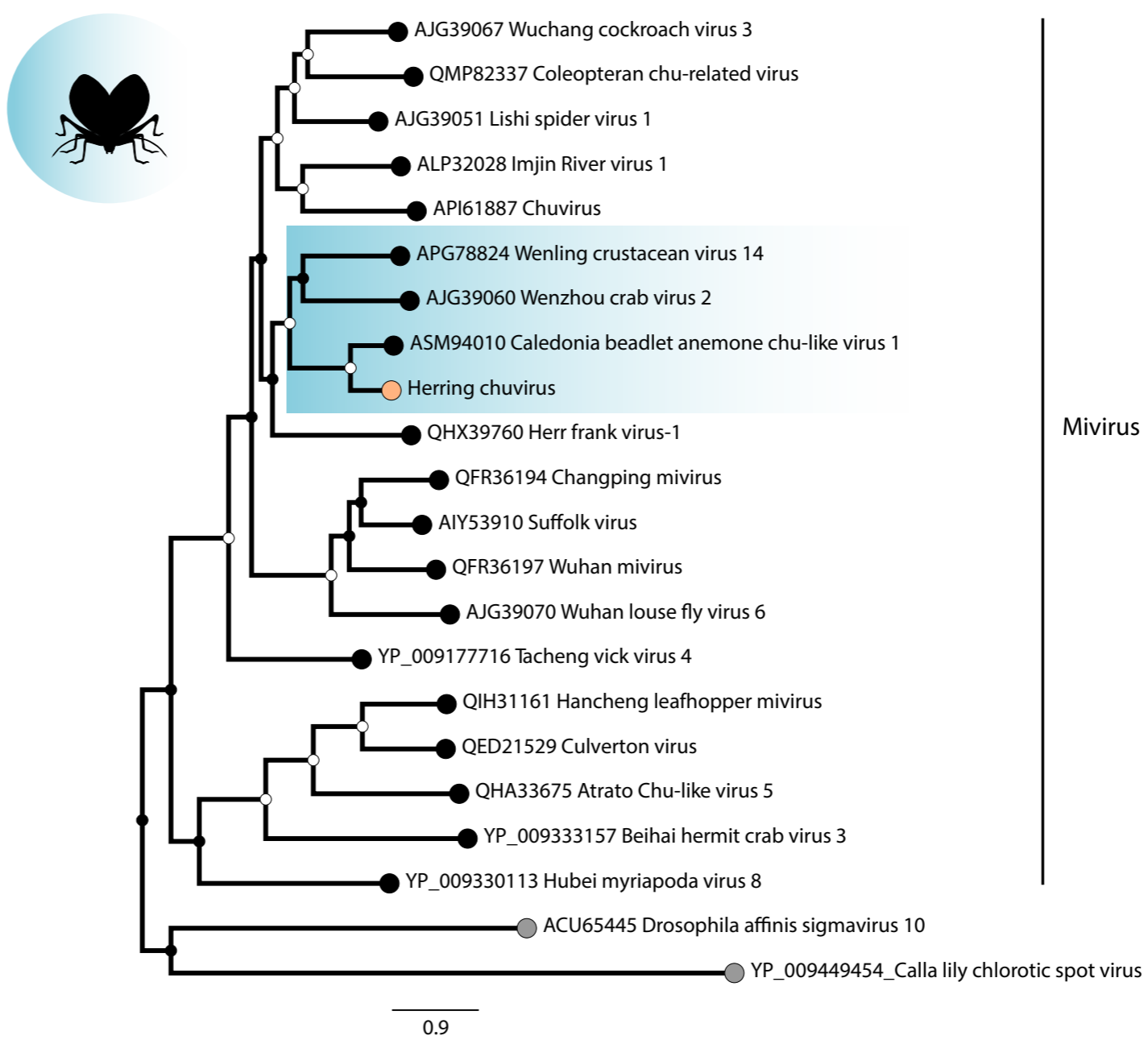

## Nodaviridae

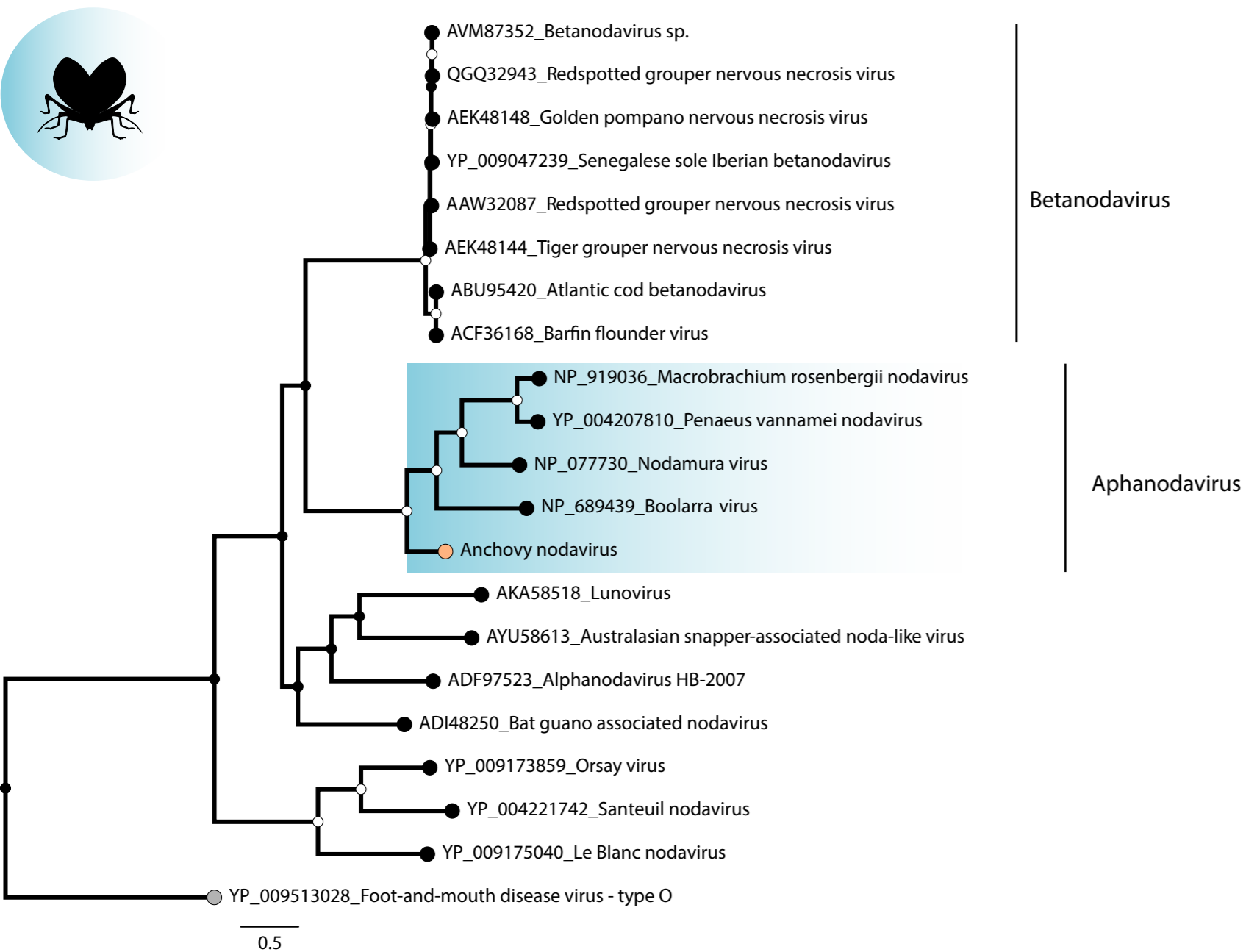

## Iflaviridae

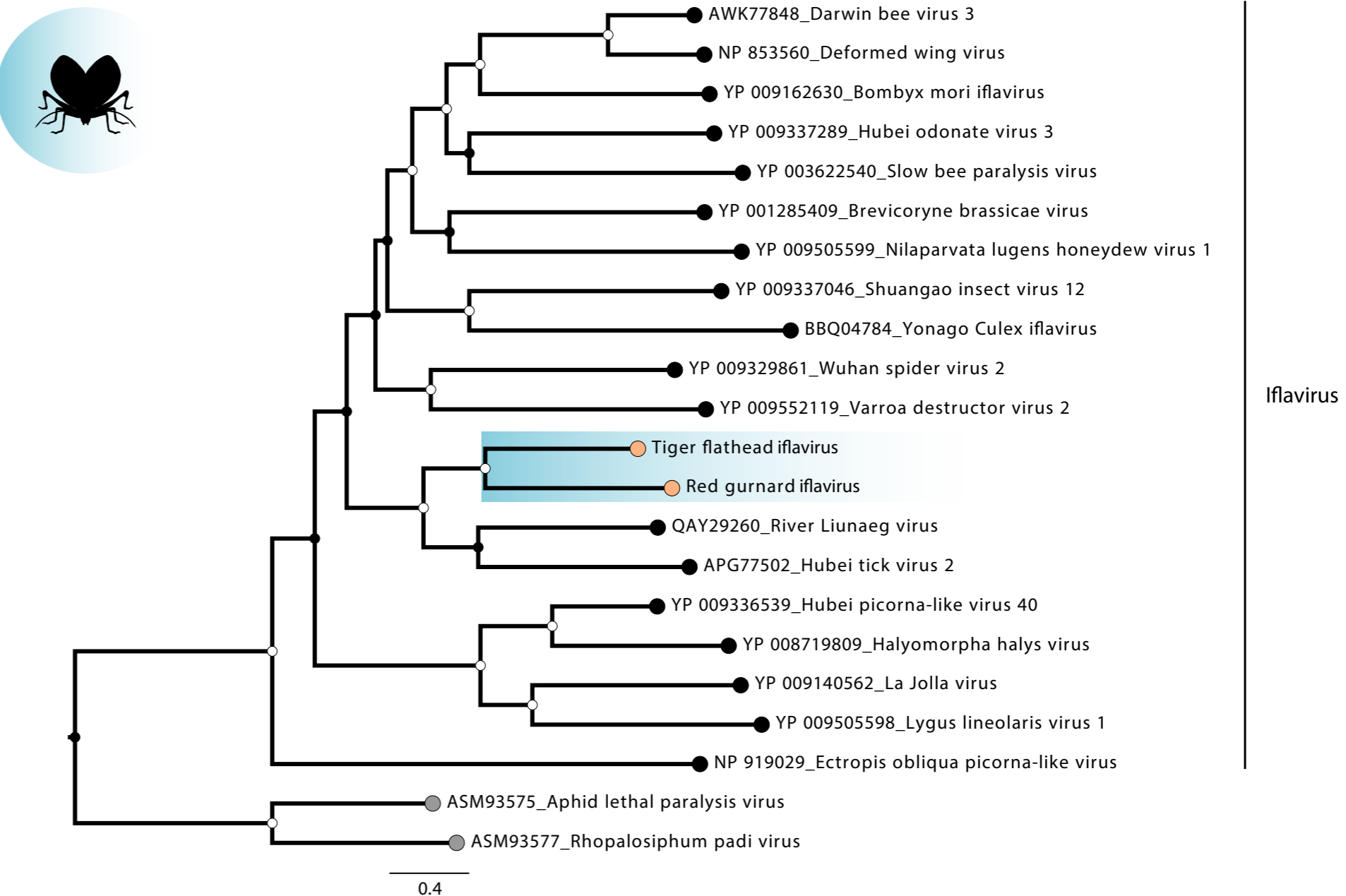

## Dicistroviiridae

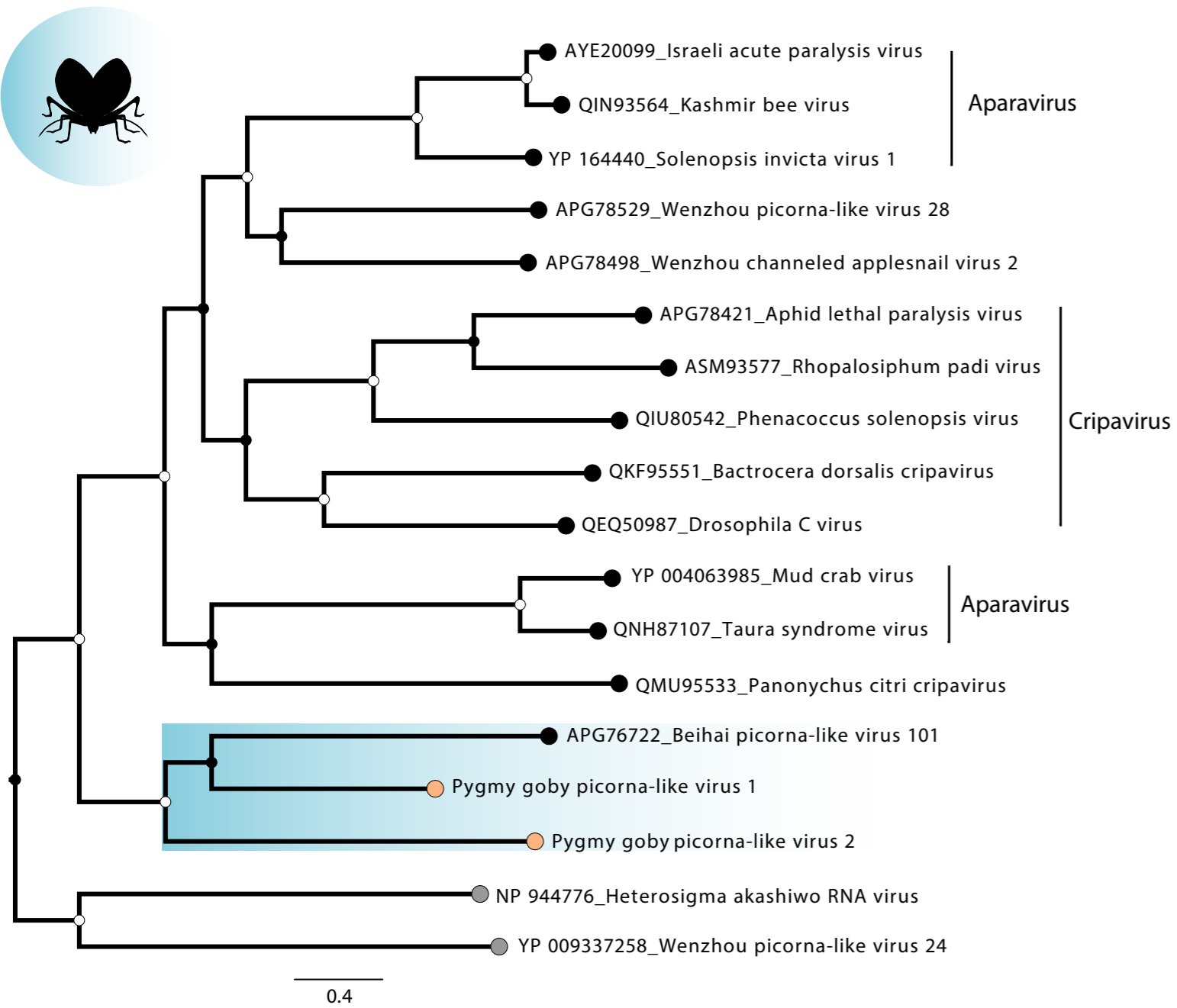

## Picornaviridae

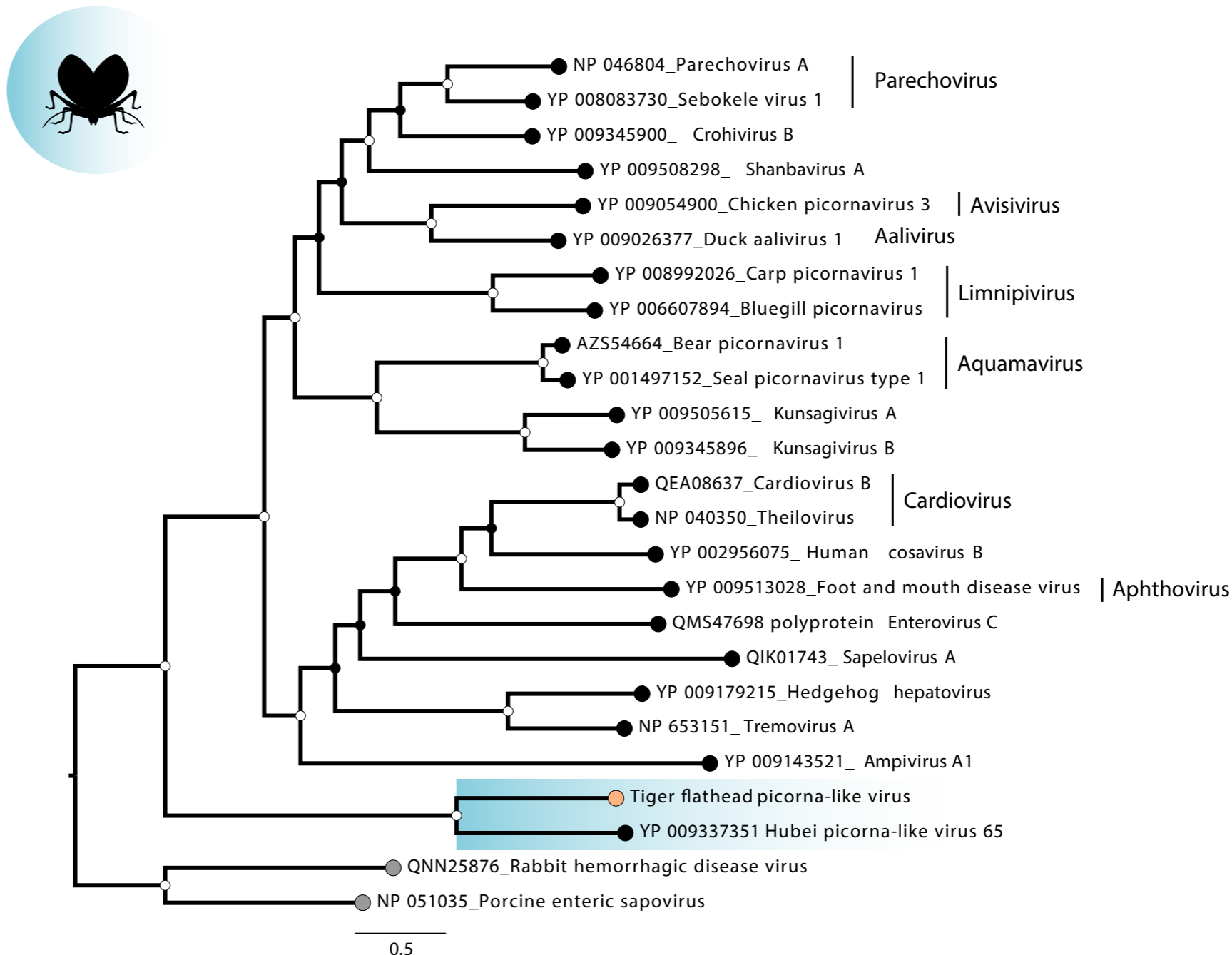

Supplement: veab005_Supplementary_Data [file veab005_supplementary_data.zip › Geoghegan.SupplementaryFigure4.pdf]
